# Prognostic Nomograms for Predicting Overall Survival and Cancer‐Specific Survival of Patients With Malignant Pheochromocytoma and Paraganglioma

**DOI:** 10.3389/fendo.2021.684668

**Published:** 2021-06-21

**Authors:** Lei Zheng, Yalong Gu, Jiangcun Silang, Jinlong Wang, Feng Luo, Baopeng Zhang, Chuanhong Li, Feng Wang

**Affiliations:** Department of Urology, People’s Hospital of Tibet Autonomous Region, Lhasa, China

**Keywords:** pheochromocytoma, paraganglioma, nomogram, survival, SEER

## Abstract

**Background:**

Malignant pheochromocytoma and paraganglioma (PPGL) are rare tumors with few prognostic tools. This study aimed to construct nomograms for predicting 3- and 5-year survival for patients with malignant PPGL.

**Methods:**

The patient data was retrieved from the Surveillance, Epidemiology, and End Results (SEER) database. A total of 764 patients diagnosed with malignant PPGL from 1975 to 2016 were included in this study. The patients were randomly divided into two cohorts; the training cohort (n = 536) and the validation cohort (n = 228). Univariate analysis, Lasso regression, and multivariate Cox analysis were used to identify independent prognostic factors, which were then utilized to construct survival nomograms. The nomograms were used to predict 3- and 5-year overall survival (OS) and cancer-specific survival (CSS) for patients with malignant PPGL. The prediction accuracy of the nomogram was assessed using the concordance index (C-index), receiver operating characteristic (ROC) curves and calibration curves. Decision curve analysis (DCAs) was used to evaluate the performance of survival models.

**Results:**

Age, gender, tumor type, tumor stage, or surgery were independent prognostic factors for OS in patients with malignant PPGL, while age, tumor stage, or surgery were independent prognostic factors for CSS (*P* <.05). Based on these factors, we successfully constructed the OS and CSS nomograms. The C-indexes were 0.747 and 0.742 for the OS and CSS nomograms, respectively. In addition, both the calibration curves and ROC curves for the model exhibited reliable performance.

**Conclusion:**

We successfully constructed nomograms for predicting the OS and CSS of patients with malignant PPGL. The nomograms could inform personalized clinical management of the patients.

## Introduction

Pheochromocytoma (PHEO) and paraganglioma (PGL) are rare neuroendocrine tumors. The annual incidence is estimated to be three cases per million (1). Among all tumors that arise from the chromaffin tissue, PHEO accounts for 80–85%, while PGL (including extra-adrenal PGL) accounts for 15–20% (2). On the other hand, the malignant PHEO and PGL cases stand at about 10, and 15–35%, respectively (3, 4). Due to lack of fundamental clinical difference between the two tumors, they are generally referred to as pheochromocytoma and paraganglioma (PPGL) tumors (5). The prognostic factors for overall survival (OS) are diverse, thus complicating accurate prediction of disease development and outcome. Nomograms have been developed and used to easily and accurately predict the survival rates of cancer patients (6). However, due to the dynamics of the PPGL, prognostic assessment tools that guide proper clinical management of the disease are limited. Here, we used the SEER database and successfully developed and verified 3- and 5-year OS and CSS prediction nomograms. The nomograms were used to assess the PPGL prognosis and guide clinical decisions in the individual management of PPGL patients.

## Patients and Methods

### Data Filtration and Extraction

PPGL patient records between 1975 and 2016 were retrieved from the SEER database using the SEER*Stat version 8.3.6 (https://seer.cancer.gov/seerstat/). The SEER database covers 34.6% of the US population, and incorporates demographic characteristics, primary tumor sites, tumor morphology, pathological diagnostic criteria, as well as treatment and follow-up records (7, 8). The cases were categorized as ICO-O-3 histology/behavior codes 8680, 8693 and 8700, respectively. Patients without positive histology examination, with secondary tumor, or with unknown tumor stage, race and survival time were excluded from the study. We randomly divided the patients into training (n = 536 [70%]) and validation (n = 228 [30%]) cohorts.

### Statistical Variables

Statistical variables included age at diagnosis, gender, race, tumor type, primary location, laterality, tumor stage, surgery, survival status, and survival duration. We categorized age as ≤30, 30–60, or >60, while race was categorized as White, Black and Others. Based on the “ICD‐O‐3 histology/behavior”, the tumors were categorized as Pheochromocytoma (PGL), Extra-adrenal paraganglioma (Extra-PGL) or Pheochromocytoma (PHEO). The primary site was used to classify as adrenal gland, aortic/carotid bodies, retroperitoneum, or others. In addition, Laterality discriminated the tumors as either unilateral or bilateral, while tumor stage was divided into localized, regional, or distant according to the SEER historic stage A (1973–2015), SEER Combined Summary Stage 2000 (2004+). As described in the Summary Stage 2018 (Version 1.7) Manual provided by SEER, localized tumors were defined as tumor confined to the adrenal gland or primary site while regional tumors were defined as involvement of Gerota’s fascia, adjacent connective tissue, invasion of adjacent organs, involvement of the regional lymph node, but without distant transfer (9). Surgery was defined as partial or total removal of the tumor.

### Statistical Analysis

Both the Kaplan–Meier method and the log-rank test were used to assess the relationship between the interrogated factors and the OS and CSS while the univariate and multivariate cox regression analysis evaluated the prognostic factors. Lasso regression was used to ensure that the models were not overfitted. Furthermore, we used the multivariable Cox proportional hazard model to construct the nomograms for the prediction of OS and CSS. In addition, C-index, calibration curves (bootstrap 1,000 resampling validation), ROC curves and computed areas under the receiver operating characteristic curve (AUC) values were used to evaluate the predictive capacity of the nomograms. Decision curve analyses (DCAs) were performed to test the clinical value of the model. The statistical analyses were performed using SPSS 26.0 (IBM Corp.) and R version 3.6.3 software (http://www.r-project.org). The statistical significance was set at *p* < .05.

## Results

### Patient Characteristics

**Figure 1:**
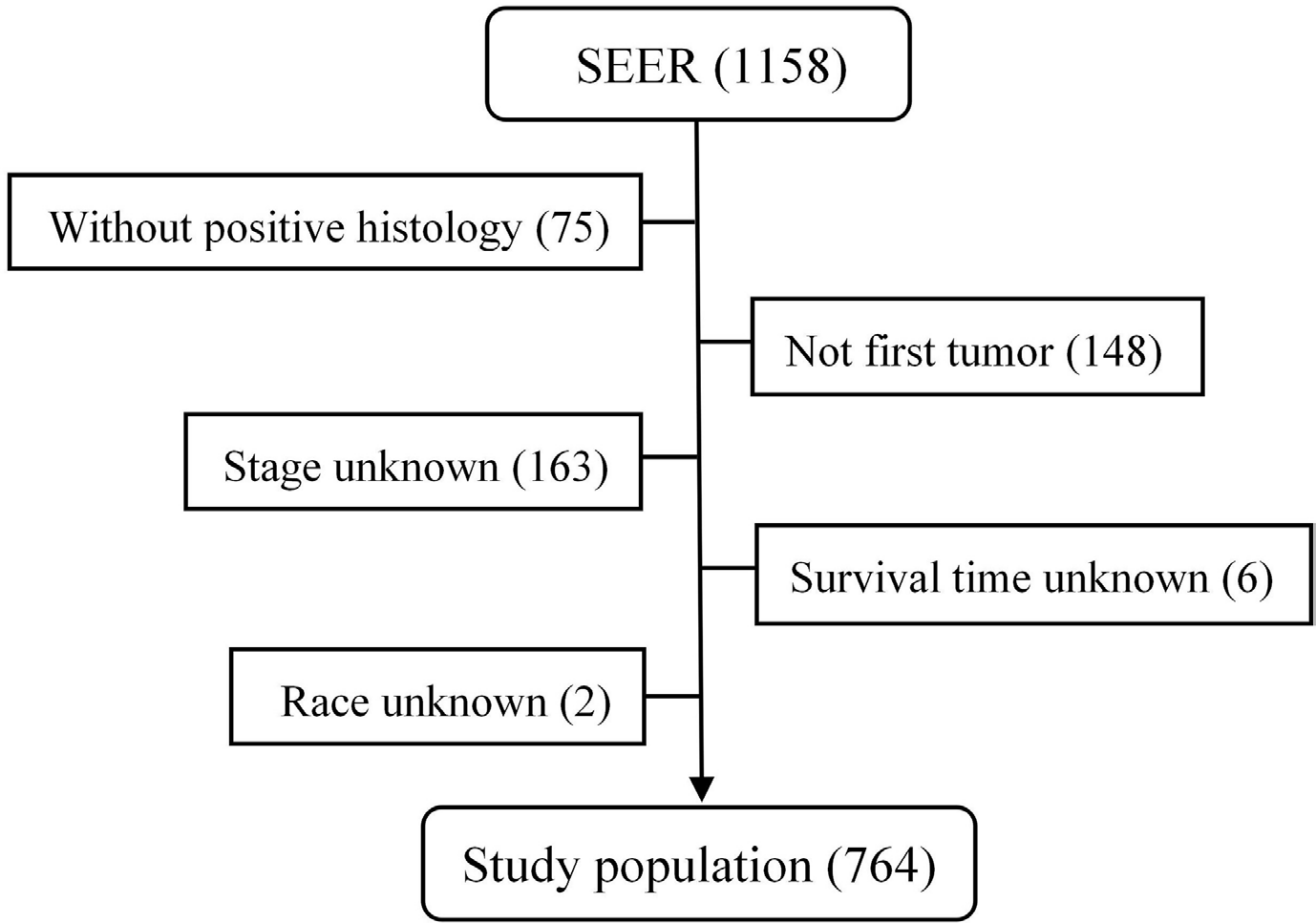
The flow chart of data process in the SEER database.

From 1975 to 2016, a total of 1,158 patients with malignant PPGL were enrolled in the SEER database. Excluding 75 patients without positive histology, 148 patients with not first tumor, 163 patients with unknown stage, six patients with unknown survival time, and two patients of unknown race, a total of 764 patients, with a median age of 49 years (7–91 years), were eligible and participated in this study (**Figure 1**). The proportion of people younger than 30, 30–60, or over 60 years old was 17.8, 55.5 or 26.7%, respectively. As shown in **Table 1**, 410 (53.7%) of the patients were male, while 354 (46.3%) were female. The overall study population was predominantly white (n = 555 [72.7%]), followed by black (n = 143 [18.7%]) and others (n = 66 [8.6%]). PHEO was the most common tumor type (n = 403 [52.7%]), followed by PGL (n = 303 [39.7%]) and Extra-PGL (n = 58 [7.6%]). A total of 375 patients (49.1%) had the primary tumor located in the adrenal gland, 148 (19.4%) located in the aortic/carotid bodies, while 81 (10.6%) patients had the tumor located in the retroperitoneum. About 160 (20.9%) patients had the primary tumor site in the other body tissues. A large proportion of the patients (96.6%) had unilateral tumors. Our data showed that 277 (36.3%) had localized tumors, while 236 (30.9%) or 251 (32.8%) had regional or distant tumors, respectively. Out of the total patients, in addition, 510 patients (66.8%) underwent surgery, 351(45.9%) died, while 257 (73.2%) were cancer-specific. For the 303 PGL patients included in the study, the total follow-up time was 437 months. Among the recurrence types, 71 (23.1%) were local, 119 (39.3%) were regional, and 114 (37.6%) were distant. Median survival was 57 months, and overall survival was 65% and 5-year survival was 48.5%. The 403 PHEO patients included in the study were followed up for 493 months, with local recurrence (194) (48.1%), regional recurrence (93) (23.1%), and distant recurrence (116) (28.8%). Median survival was 67 months, and overall survival was 66.5% and 5-year survival was 53.8%.

**Table 1:** Patient characteristics and 3- and 5-year OS and CSS rates.

characteristics	Patients		Overall survival rate (%)		P	Cancer-specific survival rate(%)		P
	No.	%	3-year	5-year		3-year	5-year	
**Age (years)**								
≤30	136	17.8	88.0 ± 2.9	83.4 ± 3.4	<0.001	88.0 ± 2.9	84.3 ± 3.3	<0.001
30–60	424	55.5	80.4 ± 2.0	71.0 ± 2.4	83.3 ± 1.9	75.2 ± 2.3		
>60	204	26.7	65.3 ± 3.4	53.0 ± 3.7	71.3 ± 3.3	61.6 ± 3.7		
**Gender**								
Female	354	46.3	80.9 ± 2.2	72.2 ± 2.5	0.023	82.4 ± 2.1	76.1 ± 2.4	0.154
Male	410	53.7	74.9 ± 2.2	64.9 ± 2.5	79.8 ± 2.1	70.7 ± 2.4		
**Race**								
White	555	72.7	77.0 ± 1.8	67.1 ± 2.1	0.288	80.1 ± 1.8	72.7 ± 2.1	0.288
Black	143	18.7	79.7 ± 3.5	71.1 ± 4.0	84.7 ± 3.2	77.4 ± 3.8		
Other	66	8.6	78.8 ± 5.2	71.5 ± 5.9	80.2 ± 5.1	72.9 ± 5.8		
**Tumor type**								
PGL	303	39.7	78.1 ± 2.5	67.3 ± 2.9	0.096	81.8 ± 2.3	72.0 ± 2.8	0.329
Extra-PGL	58	7.6	87.2 ± 4.5	76.6 ± 6.0	89.0 ± 4.3	80.4 ± 5.6		
PHEO	403	52.7	76.0 ± 2.2	67.8 ± 2.5	79.1 ± 2.1	73.0 ± 2.4		
**Primary location**								
Adrenal gland	375	49.1	76.2 ± 2.3	68.4 ± 2.5	0.280	79.1 ± 2.2	73.3 ± 2.4	0.320
Aortic/carotid bodies	148	19.4	84.0 ± 3.1	70.9 ± 4.2	88.1 ± 2.8	74.4 ± 4.1		
Retroperitoneum	81	10.6	80.3 ± 4.6	66.5 ± 5.7	81.7 ± 4.4	69.3 ± 5.5		
Other	160	20.9	74.1 ± 3.6	66.1 ± 3.9	78.5 ± 3.4	73.6 ± 3.7		
**Laterality**								
Unilateral	738	96.6	78.1 ± 1.6	68.5 ± 1.8	0.399	81.3 ± 1.5	73.5 ± 1.8	0.201
Bilateral	26	3.4	66.3 ± 9.8	61.6 ± 10.2	70.5 ± 9.5	65.5 ± 10.0		
**Tumor stage**								
Localized	277	36.3	91.8 ± 1.7	84.6 ± 2.4	<0.001	94.8 ± 1.4	90.6 ± 1.9	<0.001
Regional	236	30.9	85.0 ± 2.4	78.7 ± 2.8	88.6 ± 2.2	83.1 ± 2.6		
Distant	251	32.8	55.4 ± 3.3	40.5 ± 3.3	58.2 ± 3.3	44.2 ± 3.5		
**Surgery**								
No	254	33.2	65.8 ± 3.1	52.7 ± 3.4	<0.001	68.9 ± 3.1	57.2 ± 3.5	<0.001
Yes	510	66.8	83.5 ± 1.7	75.7 ± 2.0	86.8 ± 1.6	80.8 ± 1.9		

HR, Hazard Ratio; CI, Confidence Interval; Extra-PGL, Extra-adrenal paraganglioma.

### Prognostic Signature for Predicting OS and CSS

**Figure 2:**
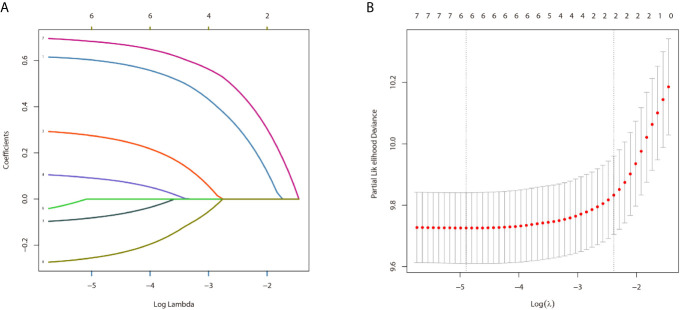
The result of the lasso regression **(A, B)**.

In the univariate analysis for OS, age (*P* < .001), gender (*P* = .016), tumor type (P = .036), tumor stage (*P* < .001) or surgery (*P* < .001) were significantly correlated with OS in PPGL patients. Similarly, multivariate analysis showed that age (*P* < .001), gender (*P* = .004), tumor type (*P* = .031), tumor stage (*P* < .001), or surgery (*P* = .004) were associated with the OS. In contrast, both univariate and multivariate analysis showed that only age (*P* = .002), tumor stage (*P* <.001), or surgery (*P* < .001) were significantly correlated with CSS in PPGL patients (**Tables 2** and **3**). To avoid overfitting, lasso regression was used to determine the above variables included in the model construction (**Figure 2**).

**Table 2:** Univariate and multivariate Cox proportional hazards regression analyses of variables associated with overall survival.

characteristics	Univariate analysis			Multivariable analysis		
	HR	95% CI	P	HR	95% CI	P
**Age (years)**						
≤30	Reference			Reference		
30–60	1.661	1.116–2.472	0.012	1.458	0.976–2.179	0.066
>60	2.843	1.863–4.338	<0.001	2.908	1.883–4.493	<0.001
**Gender**						
Female	Reference			Reference		
Male	1.371	1.06–1.773	0.016	1.477	1.134–1.924	0.004
**Race**						
White	Reference			Not included		
Black	0.748	0.525–1.066	0.108			
Other	0.830	0.533-1.293	0.410			
**Tumor type**						
PGL	Reference			Reference		
Extra-PGL	0.537	0.301–0.959	0.036	0.717	0.399–1.289	0.266
PHEO	1.006	0.773–1.309	0.964	1.368	1.028–1.819	0.031
**Primary location**						
Adrenal gland	Reference			Not included		
Aortic/carotid bodies	0.925	0.646–1.324	0.671			
Retroperitoneum	0.991	0.673–1.457	0.962			
Other	0.960	0.688–1.339	0.808			
**Laterality**						
Unilateral	Reference			Not included		
Bilateral	0.925	0.456–1.877	0.830			
**Tumor stage**						
Localized	Reference			Reference		
Regional	1.135	0.797–1.617	0.483	1.277	0.888–1.836	0.186
Distant	4.042	2.963–5.514	<0.001	4.038	2.908–5.608	<0.001
**Surgery**						
No	Reference			Reference		
Yes	0.477	0.364–0.625	<0.001	0.644	0.478–0.869	0.004

HR, Hazard Ratio; CI, Confidence Interval; Extra-PGL, Extra-adrenal paraganglioma.

**Table 3:** Univariate and multivariate Cox proportional hazards regression analyses of variables associated with cancer-specific survival.

Characteristics	Univariate analysis			Multivariable analysis		
	HR	95% CI	P	HR	95% CI	P
**Age (years)**						
≤30	Reference			Reference		
30–60	1.605	1.029–2.502	0.037	1.442	0.919–2.263	0.112
>60	2.173	1.337–3.532	0.002	2.210	1.355–3.603	0.001
**Gender**						
Female	Reference			Not included		
Male	1.263	0.942–1.695	0.119			
**Race**						
White	Reference			Not included		
Black	0.688	0.453–1.046	0.080			
Other	0.754	0.443–1.284	0.299			
**Tumor type**						
PGL	Reference			Not included		
Extra-PGL	0.554	0.287–1.072	0.080			
PHEO	0.962	0.287–1.072	0.800			
**Primary location**						
Adrenal gland	Reference			Not included		
Aortic/carotid bodies	1.011	0.673–1.517	0.959			
Retroperitoneum	1.230	0.807–1.874	0.336			
Other	0.918	0.620–1.361	0.671			
**Laterality**						
Unilateral	Reference			Not included		
Bilateral	1.002	0.443–2.266	0.995			
**Tumor stage**						
Localized	Reference			Reference		
Regional	1.490	0.954–2.329	0.080	1.502	0.960–2.350	0.075
Distant	6.019	4.088–8.863	<0.001	5.319	3.562–7.941	<0.001
**Surgery**						
No	Reference			Reference		
Yes	0.411	0.305–0.555	<0.001	0.618	0.450–0.850	0.003

HR, Hazard Ratio; CI, Confidence Interval; Extra-PGL, Extra-adrenal paraganglioma.

### Survival Analyses

**Figure 3:**
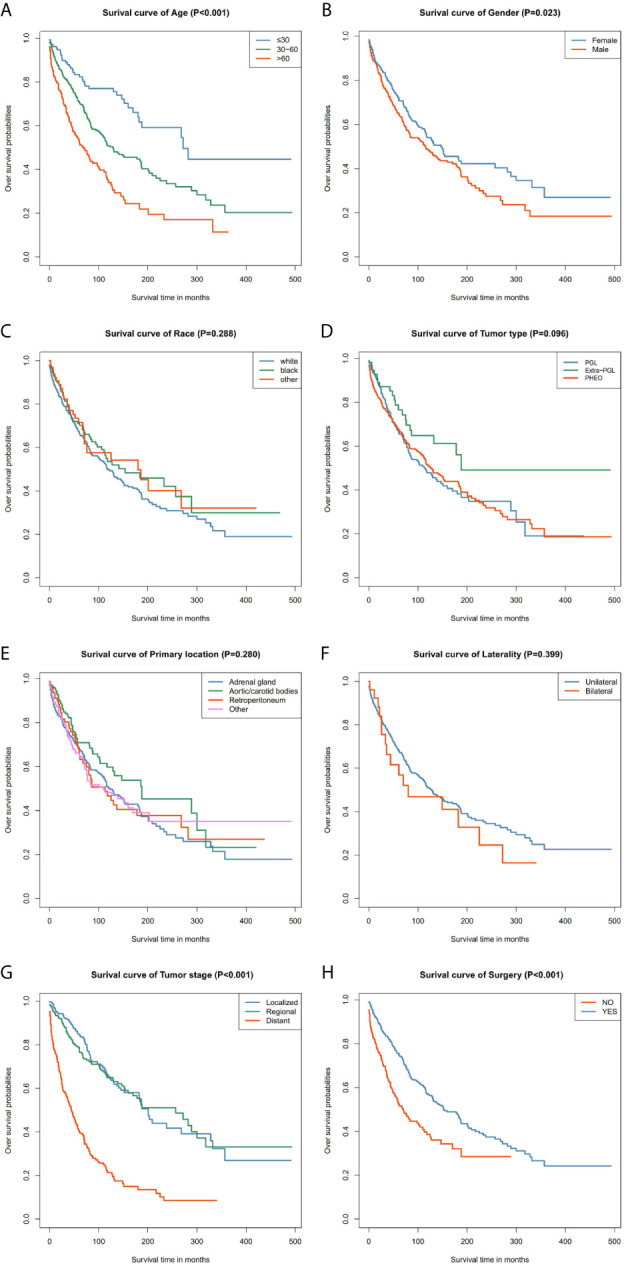
Kaplan–Meier curves of overall survival for patients based on: **(A)** age; **(B)** gender; **(C)** race; **(D)** tumor type; **(E)** primary location; **(F)** laterality; **(G)** tumor stage; **(H)** surgery.

**Figure 4:**
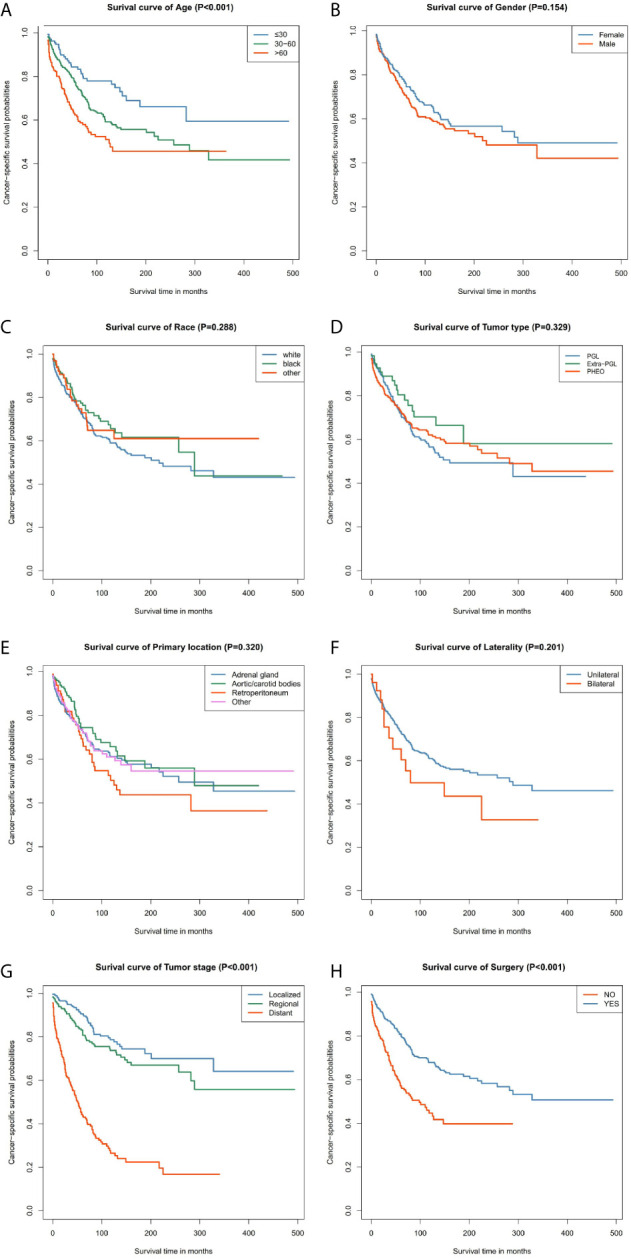
Kaplan–Meier curves of cancer-specific survival for patients based on: **(A)** age; **(B)** gender; **(C)** race; **(D)** tumor type; **(E)** primary location; **(F)** laterality; **(G)** tumor stage; **(H)** surgery.

Our data demonstrated that patients older than 60 years have poor prognosis. However, unlike their male counterparts, female patients had better disease prognosis, thus longer OS. The survival rate of patients with distant tumor staging was significantly lower than that of patients with localized or regional tumor. In addition, patients who had undergone surgery had better OS and CSS. There was no significant difference between the OS and CSS in race, tumor type, primary location, or laterality (**Figures 3** and **4**).

### Nomogram Construct and Validate

**Figure 5:**
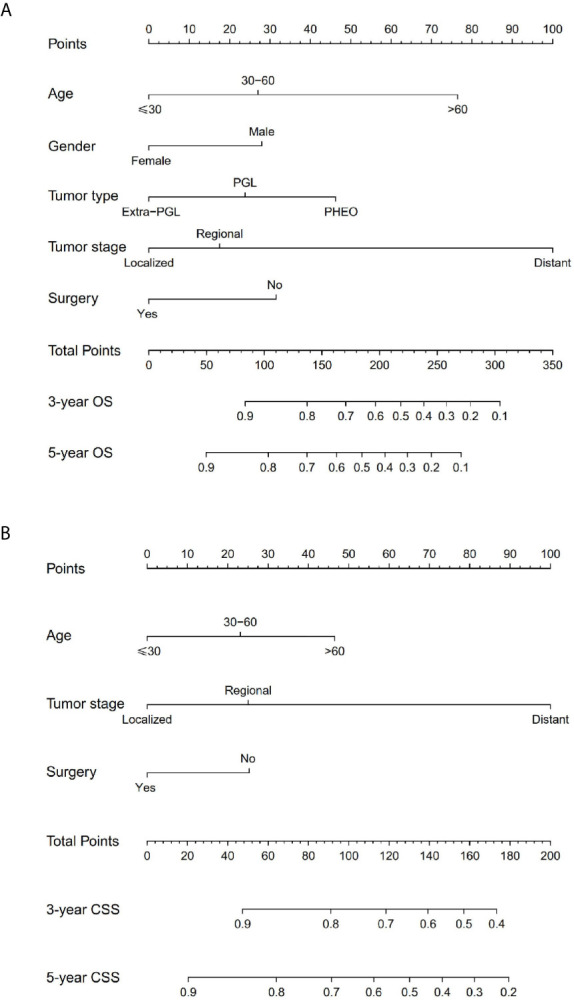
Nomograms predicting 3- and 5-year OS **(A)** and CSS **(B)** of patients with malignant PPGL.

**Figure 6:**
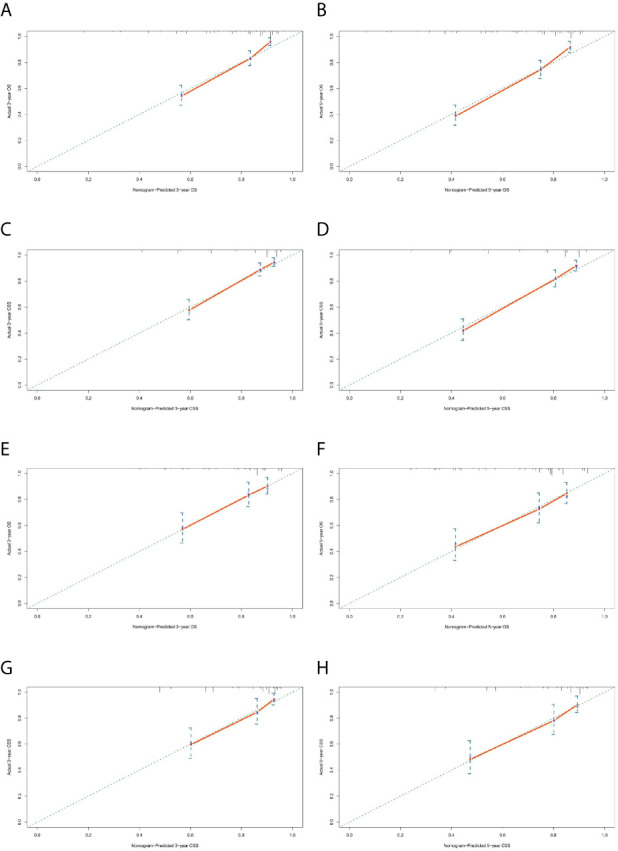
Calibration charts of the nomograms for 3- and 5-year OS and CSS prediction of the training cohort **(A–D)** and validation cohort **(E–H)**.

**Figure 7:**
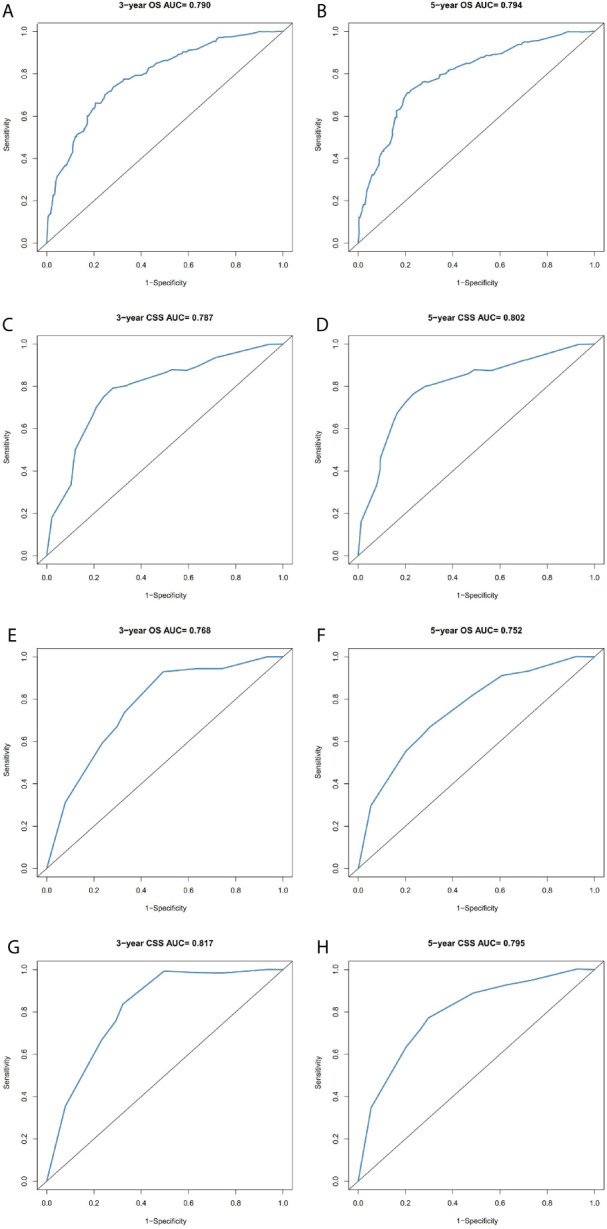
Comparison of the ROC curves of the nomograms for 3- and 5-year OS and CSS prediction of the training cohort **(A–D)** and validation cohort **(E–H)**.

**Figure 8:**
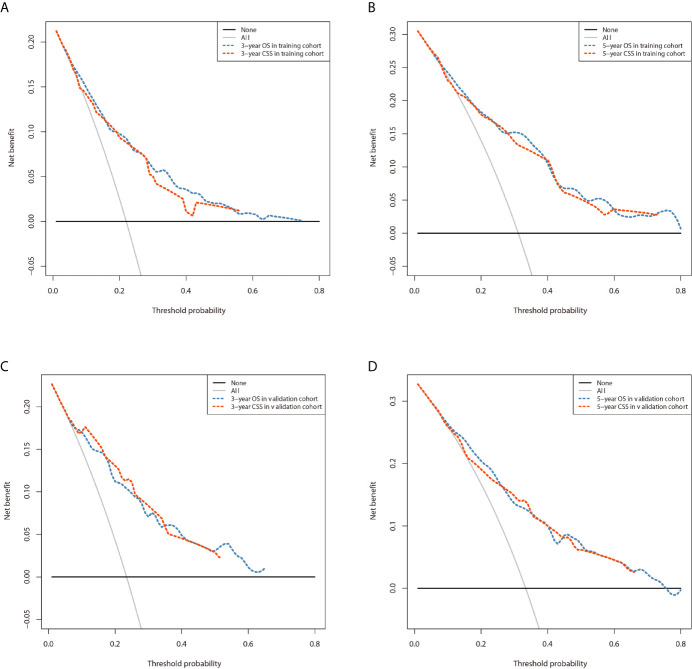
Comparison of the DCA curves of the nomograms for 3- and 5-year OS and CSS prediction of the training cohort **(A, B)** and validation cohort **(C, D)**.

The prominent risk factors determined by the multivariate analysis were applied to construct nomograms that could predict the 3- and 5-year OS and CSS for PPGL (**Figure 5**). We used the scale at the top of each nomogram to give a point to each prognostic variable and the proportions at the bottom of each nomogram (adding up the points of all variables) to predict the 3- and 5-year survival rates. The nomogram used for OS prediction showed that prognosis was mainly affected by the tumor stage followed by age, tumor type, surgery and gender. Similarly, the nomogram for CSS indicated that tumor stage was also the most significant factor affecting prognosis, followed by age and surgery. Besides, the nomogram was verified by the C-index, calibration curve or ROC curves (**Figures 6** and **7**). The C-indexes of the training cohort were 0.747 and 0.742 for OS and CSS, respectively, while the validation cohort had 0.712 and 0.741 for OS and CSS, respectively. The 3- and 5-year AUC values of the training cohort (for OS: 0.790, 0.794; and for CSS: 0.787, 0.802), while the validation cohort (for OS: 0.768, 0.752; and for CSS: 0.817, 0.795). All the calibration curves for the model exhibited satisfactory performance. The 3- and 5-year DCA curves indicated that both models generated net profits in the training and validation cohorts (**Figure 8**).

## Discussion

PPGL is a rare disease with relatively low incidence. Currently, only a variety of pathological indicators can be used to predict the malignant behavior of PPGL, and there is no tool to predict prognosis and guide follow-up. Lack of data from large randomized prospective studies on the PPGL tumor hampers proper clinical management. In 2017, and for the first time, the American Joint Committee on Cancer (AJCC) released the 8th edition of the TNM staging system, which included PPGL (10). But the TNM staging system alone is incapable of accurately assessing the prognosis of the PPGL patients. The World Health Organization (WHO) defines malignant PPGL as metastasis of non-chromaffin sites (liver, bone, lung, kidney, lymph nodes, etc.) far away from the primary tumor, rather than local invasion. Unlike many other tumors, there is no histological evidence, molecular, or genetic markers that can robustly determine the severity of PPGL (11). Tumor metastases in non-endocrine tissues are the only recognized standard for the diagnosis of malignant PPGL (12, 13).

The SEER database covers 34.6% of the US population and present s a reliable resource for studying malignant tumors with low incidence. At present, all PPGLs are considered to be potentially malignant due to the genetic heterogeneity and the complexity of pathogenesis. Currently, there is no clear pathological classification to distinguish patients with malignant PPGL. But should the same follow up be followed for all of these patients? Probably not. According to the clinical prediction model established by us, the prognosis of patients with PPGL can be predicted and individualized follow-up plan can be formulated. We constructed 3- and 5-year OS and CSS nomograms, and verified their predictive performance, thus enhancing personalized treatment and survival assessment. The OS prediction model comprises of age, gender, tumor type, tumor stage, and surgery, while the CSS prediction model consists of age, tumor stage, and surgery. The most significant indicator on the patients’ OS and CSS in both models was the tumor stage. Compared to CSS, age at diagnosis had a greater impact on the OS. According to the Kaplan–Meier curve and log-rank analyses, most of the patients were over 30 years old, and the prognostic prediction efficiency on survival declines with the increase in age. Currently, there is inconsistent data on the role of gender in PPGL outcomes. Some studies have shown that gender is an independent risk factor (14), while others have shown that gender does not play a significant role in the disease outcome (15). In our study, the female patients indicated better OS, which was consistent with the previous reports (16, 17). The discrepancy was not indicated to be an independent factor in the multivariate analysis of CSS. Therefore, gender is an independent prognostic factor and has a distinct effect on the OS of patients with PPGL. Overall, this study has given insight and laid the foundation for further interrogation of the relationship between demographic features and disease prognosis. Besides, our study found that PPGL was highly diverse in the clinical course. Compared with the PHEO or PGL, Extra-PGL showed better OS, but moderate CSS. In addition, whereas the tumor primary location has been shown to be an independent risk factor (18), our data demonstrated no statistical significance in OS and CSS.

PHEO and PGL have different tumor behaviors. The term pheochromocytoma is now reserved for intra-adrenal tumors, while a similar tumor outside the adrenal medulla is defined as an extra-adrenal paraganglioma and is further named after its anatomical origin. The largest collection of parasympathetic parasympathetic ganglia is the carotid body of the head and neck. Unlike pheochromocytoma, many extra-adrenal paragangliomas usually have no obvious clinical symptoms. Although sympathetic paragangliomas are usually functional, less than 4% of head and neck paragangliomas present symptoms associated with excessive catecholamine secretion. Therefore, many parasympathetic paragangliomas are incidentally discovered by imaging or present as slow-growing, painless masses. Through analysis of the SEER database, patients with PHEO had higher 5-year and overall survival rates than those with PGL. In terms of tumor invasion, patients with PGL are more likely to have regional and distant metastasis, while patients with PHEO are mainly subject to local metastasis (2, 10, 13).

The treatment of malignant pheochromocytoma has not greatly changed in recent years, and surgical resection remains the primary option (19, 20). Surgery excises primary tumors and removes local and distant metastases. However, malignant tumors with distant metastases and recurrence prove difficult for surgical excision (21, 22). Partial tumor resection, also known as cytoreductive surgery, is another option, which can alleviate clinical symptoms (tumor compression, hypertension, and plasma catecholamines level), and mitigate the immunosuppressive effects of the tumors (23). However, the survival advantage of the cytoreductive surgery has not been demonstrated in random clinical trials (24). In our study, we evaluated the effect of surgery (including radical and partial resection) on OS and CSS in patients with PPGL. This is consistent with the previous studies (14). However, the corresponding effect requires combination with other treatments (25). Clinical follow-up of patients with PPGL is recommended throughout life (16).

With the advancement of medicine and a deeper understanding of pheochromocytoma, molecular biology has been applied in the treatment of PPGL in recent years and demonstrated remarkable effect (26, 27). It is widely held that at least 30–40% of PPGL are induced by inherited genes (28, 29). Mutations in the succinate dehydrogenase subunit B (SDHB) gene are the most common genetic alteration in PPGL (2, 30). In another cross-database study, specifically targeting malignant PPGL cohorts, oncogenic mutations were identified in seven of 13 patients (53.8%), with the most common mutation located at ATR X, 15.4% (14). The unique genetic background of malignant PPGL may be involved. Besides, the molecular markers are closely related with virulence, metastasis, and therapy (31). Due to the genetic heterogeneity and the complexity of pathogenesis of PPGL, the targeting drugs for malignant PPGL are still in the research phase, so it is difficult to assess the genetics of PPGL. Comprehensive patient assessment is critical when considering genetic diagnosis, as clinical manifestations as well as radiological and biochemical features will guide clinicians to make a correct diagnosis (32). Therefore, to establish more effective prediction systems, future research will combine both molecular markers and other clinical risk factors. Our study, however, was constructed the nomograms based on retrospective data from the SEER database, thus might lack detailed patient records (33). For instance, tumor size was not recorded in the SEER database before 2004. Besides, the tumor markers, gene mutations and other information are not available from the database. Therefore, these factors were not included in the development of the final model. In particular, due to the limitations of the SEER database, this nomogram is not generalizable across all ages/ethnicities/genetic syndromes. Whereas we validated the nomograms by C-index, calibration curve, or ROC curve, prospective studies are needed for external verification to obtain more reliable nomograms.

## Conclusion

In conclusion, we have successfully established nomograms for the prediction of 3- and 5-year OS and CSS in patients with PPGL. The nomogram proved to be accurate and reliable in the prediction of the disease outcome. The nomogram can be used as an independent tool to assess the prognosis and guide personalized follow-up for clinical management of malignant PPGL patients.

## Data Availability Statement

The datasets presented in this study can be found in online repositories. The names of the repository/repositories and accession number(s) can be found in the article/**Supplementary Material**.

## Ethics Statement

Ethical review and approval was not required for the study on human participants in accordance with the local legislation and institutional requirements. Written informed consent for participation was not required for this study in accordance with the national legislation and the institutional requirements.

## Author Contributions

ZL and SC performed design the study and edited the manuscript. ZP, LF and WL were responsible for confirming research content, data filtration and extraction of this study.WF and LH performed review and quality control of this study. ZL and GYL were major contributors in studies statistical analyses, nomogram development and validate. All authors contributed to the article and approved the submitted version.

## Conflict of Interest

The authors declare that the research was conducted in the absence of any commercial or financial relationships that could be construed as a potential conflict of interest.
